# Participative Facility Planning for Obstetrical and Neonatal Care Processes: Beginning of Life Process

**DOI:** 10.1155/2016/7836493

**Published:** 2016-12-06

**Authors:** Jori Reijula, Sauli Karvonen, Hanna Petäjä, Kari Reijula, Liisa Lehtonen

**Affiliations:** ^1^Finnish Institute of Occupational Health, Neulaniementie 4, 70210 Kuopio, Finland; ^2^SKA-Research Oy, Teollisuustie 9, 02880 Veikkola, Finland; ^3^Turku University Hospital, Kiinamyllynkatu 4-8, 20520 Turku, Finland; ^4^University of Helsinki, Yliopistonkatu 4, 00100 Helsinki, Finland

## Abstract

*Introduction.* Old hospitals may promote inefficient patient care processes and safety. A new, functionally planned hospital presents a chance to create an environment that supports streamlined, patient-centered healthcare processes and adapts to users' needs. This study depicts the phases of a facility planning project for pregnant women and newborn care processes (beginning of life process) at Turku University Hospital.* Materials and Methods.* Project design reports and meeting documents were utilized to assess the beginning of life process as well as the work processes of the Women's and Children's Hospital.* Results.* The main elements of the facility design (FD) project included rigorous preparation for the FD phase, functional planning throughout the FD process, and setting key values: (1) family-centered care, (2) Lean thinking and Lean tools as the framework for the FD process, (3) safety, and (4) cooperation.* Conclusions.* A well-prepared FD project with sufficient insight into functional planning, Lean thinking, and user-centricity seemed to facilitate the actual FD process. Although challenges occurred, the key values were not forgone and were successfully incorporated into the new hospital building.

## 1. Introduction

Hospital patient care processes are often forced to adapt to impractical facilities. The premises used for different functions of the same process can be scattered around the hospital, a far cry from seamless patient care. Planning a new hospital presents a unique opportunity to address this issue [1].

Functional planning should guide the design of a new hospital [2] and aim to create facilities that support efficient, streamlined, and patient-centered healthcare (HC) processes while avoiding silos [3]. Functional planning for a new hospital starts with a critical review of current processes in order to modify and develop them so that they will function effectively in the new facilities [4]. There should also be goals for improving the quality, safety, and cost-efficiency of treatment and the work environment [5], as well as the wellbeing and satisfaction of users [6].

At Turku University Hospital (H1), the need for a new hospital building was identified. The facilities at H1, which was planned in the 1950s and built in 1968, were becoming obsolete: there were operation bottlenecks, problems with the quality of indoor air, frequent sewage breakdowns, and fire safety issues [7]. Renovation of the old facilities would have stipulated a temporary hospital for the duration of the work. In the case of renovation, the existing tall, narrow, outdated building structure would have set many limitations for creating a functional, modern hospital [8]. Therefore, a planning process was launched to build a new hospital called the Women's and Children's Hospital. One of its core processes was to be the care of pregnant women and newborns. As this process combines obstetrical and neonatal care, it was reasonable to locate all gynecologic and pediatric care in the same building. The group responsible for functional planning related to the care of pregnancies, deliveries, and newborns was called The Beginning of Life Group. It included all antenatal and perinatal care, newborn care, neonatal intensive care, the breast milk bank, and the follow-up clinic for very preterm infants. The increasingly popular* Lean thinking* was an integral framework for the functional planning of the process (see [9–11] for more information on Lean HC).

This study aims to depict the formula of functional facility planning carried out by The Beginning of Life Group at H1. The aim is to illuminate the different phases of the facility planning process as well as the most remarkable challenges encountered.

## 2. Materials and Methods

### 2.1. Case Hospital

H1 is one of five university hospitals in Finland and covers a population of approximately one million Finnish citizens. The Beginning of Life Group was led by a pediatrician and included 17 other members: one obstetrician, six midwives, five nurses, one anesthetist, one radiologist, one pharmacist, one patient representative (a parent of two preterm infants, so representing parents and patients), and a project coordinator.

### 2.2. Analysis Method

Three researchers analyzed design reports and meeting documents in order to assess the beginning of life process. The current structure of the process was identified and depicted using a particular patient flow analysis and value stream mapping. The patient flow analysis focuses on describing patients' physical movements in the hospital [12, 13]. Value stream mapping, a well-known Lean technique, does not describe physical movements of patients precisely but instead focuses on information flow modelling. It also includes lead time information [14]. Thus, the management method used in the planning process is a combination of Lean management and patient flow analysis. Moreover, potentials for synergy, but also bottlenecks, functional problems, and other forms of waste in the process were identified using root cause analysis.

## 3. Results

### 3.1. The Current Care Process in the Old Hospital Building

**Figure 1:**
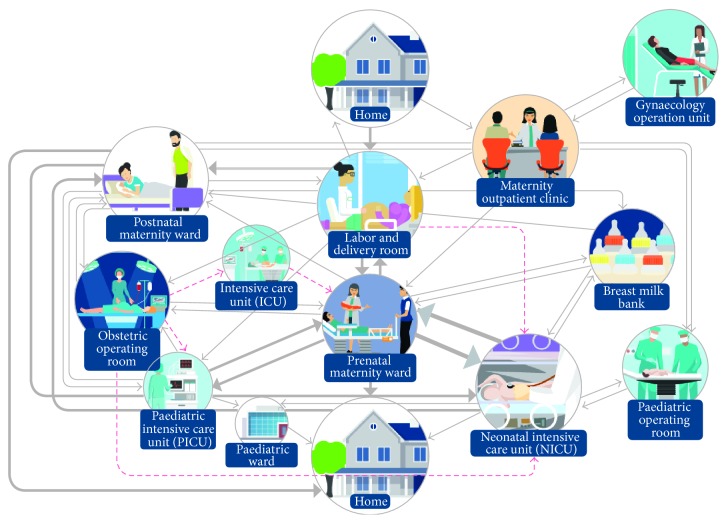
The old H1 patient flow process model for the care of pregnant women and newborns. The arrows demonstrate patient transfers between H1 units. The girth of the line delineates the volume of patient flow, with a thick line denoting an intense flow. Critical patient transfers are marked with a dotted red line [15].

The structure of H1 was identified as outdated. The hospital layout poorly supported current work processes, which had been revised in the decades since the old hospital was built. Due to the suboptimal hospital layout, work processes were dispersed across several units of the hospital on four different, but not adjacent, floors. In addition, the elevators were commonly sluggish. This caused unnecessary waiting and back-and-forth transfers of patients from one unit to another (see Figure 1). The patient flow in H1 was far from streamlined, as patients were obliged to make several trips between units during a single hospital visit (see Figure 1). Furthermore, the distance (on average approximately 100 meters) from the delivery room to the neonatal intensive care unit (NICU) was a threat to patient safety as the most ill newborns, after initial resuscitation, had to be transferred seven floors up by elevator and then to the other end of the long building through several doors and over several doorsteps. The reason for the delivery rooms and the NICU not being in close proximity of each other is because there was no modern neonatal intensive care when the old hospital was built.

The insufficient number of patient rooms (12) in the labor and delivery unit caused unnecessary waiting for both mothers and the nursing staff. Only one delivery room in the labor ward had a birth pool, and mothers took turns using the bathing facilities during labor contractions. The lack of space in the delivery rooms and the electric cords lying on the floor presented safety concerns. This was exacerbated by the lack of HC personnel.

The area in the delivery room used for newborn resuscitation and monitoring was small. Therefore, the newborns requiring longer observation or procedures after initial resuscitation were transferred to the NICU, leading to frequent NICU admission.

The mothers of infants cared for at the NICU stayed in the prenatal maternity ward. This caused frequent daily traffic as mothers visited their infants several times a day and returned to their ward for care and meals. After NICU care, as soon as they were stable, over 60% of infants were transferred to the postnatal maternity ward so that mother-infant separation was minimized. Ideally, maternal care and parent beds would be located in the NICU to avoid a large number of within-hospital transfers. In most cases, the limited number of family rooms in the postnatal maternity ward prevented the overnight presence of fathers.

### 3.2. Predesign Phase

The groundwork for the functional design was carried out years before the actual process was initiated. In 2001, a lifecycle decision was made regarding the old H1 Women's and Children's Hospital. It was concluded that the facilities were outdated and needed to be renovated or replaced. However, no official decision was made until 2011, when hospital replacement was agreed upon and the functional design process officially commenced.

Before the actual design process commenced, however, the Department of Pediatrics and the Department of Obstetrics and Gynecology held several joint meetings about their visions for a new hospital. Held between 2005 and 2009, these meetings generated reports about the projected needs of the new hospital space. Key personnel from the Department of Pediatrics and the Department of Obstetrics and Gynecology visited new perinatal centers and centers undergoing renovations or building processes in the United States (e.g., Rainbow Babies and Children's Hospital, Cleveland, Ohio: a three-year work period in 1997–2000 followed by several visits until 2011), Canada (e.g., Sunnybrook Hospital, Toronto: a visit in 2011), and Sweden (e.g., Uppsala University Hospital: several visits from 2005 and a six-month work period in 2011) to gain insight into state-of-the-art solutions. Key personnel acquainted themselves with the planning processes of these international sites. One crucial observation was that a certain number of key fundamentals should be clearly and thoroughly determined prior to the facility design (FD) process and prioritized during the process. Furthermore, information sharing between hospitals was seen as pertinent. Systematic participation in HC FD forums (e.g., Graven's Conference on the Physical and Developmental Environment of the High Risk Infant) also provided essential knowledge about other hospitals' state-of-the-art innovations and whether they had been good solutions from the users' perspective or nonoptimal solutions which had led to bottlenecks and other unintended functional problems.

### 3.3. Design Phase

A multidisciplinary planning group (*n* = 17) was founded in 2011 and called The Beginning of Life Group. Its main focus was on developing a functional plan for the beginning of life process including pregnancy, labor, and newborn care. The planning group held approximately 20 design meetings. H1 administration attempted to allocate up to 30% of key personnel's work time for the FD project to cover a six-month period.

The Beginning of Life Group started by discussing the main values that would guide the planning process. Four values were chosen based on the group discussion (Table 1):

1. Family-centered care was seen as the top priority for the FD. It was operationalized as minimizing parent-infant separation. The new hospital environment was planned to support parents and newborn infants staying together for the entire treatment period. It emphasized the customer point-of-view and attempted to improve user-centricity according to the strategy of the hospital district. Family rooms provide privacy, thereby increasing the comfort of parents and their overnight presence in the hospital.
2. The group made Lean fundamentals the visible framework for the design phase: eight types of waste in HC (defined in Lean literature) were systematically sought and eliminated. Reducing the number of patient transfers within and between units was an important strategy to eliminate unnecessary work steps (transferring patients and information, signing in and out, and cleaning rooms between patients). Another strategy was to decrease the travel distance when transferring sick newborns from the delivery room to the NICU. Flexible, modifiable rooms were planned for the NICU that could accommodate the changing needs of a patient throughout their hospital stay (usually beginning with more intensive care and then decreasing in intensity over time).
3. The group aimed to improve patient and staff safety by various means: effort was made to develop disturbance-free treatment periods, such as fewer transfers within and between units, thereby decreasing communication errors by the staff and minimizing hazards related to the transfer of sick patients. Moreover, single family rooms isolated the patients and their relatives from other patients, decreasing the likelihood of spreading infections.
4. The group named enhanced cooperation as one method for breaking the boundaries of old silos and reorganizing care based on patient care pathways. This was pursued by creating a single process with a common goal: getting the mother and baby home healthy. The new environment would decrease boundaries between units, even including a stabilization room for newborns in the delivery room where both neonatal nurses and midwives would work together. Midwives would provide care for mothers and their infants in the NICU. The physical proximity of the prenatal maternity ward to the labor and delivery room and the NICU aimed to enhance synergy, cooperation, and communication between the staff of these units.

A parent representative was invited to take part in The Beginning of Life Group so that patient and parent perspectives were taken into account. The representative held discussions with a larger group of parents in a peer-support group in order to bring their views into the planning process.

Information gathered from the staff and secretaries regarding supportive functions proved useful to the design group.

The planning group assessed patient and information flows in order to avoid unnecessary traffic within the hospital and to increase the efficiency of the units. Tools such as value stream mapping were utilized for a patient flow analysis, starting from pregnant women and caesarean sections through to neonatal intensive care and transitions to home. Bottlenecks, functional problems, other forms of waste in the process, and potentials for synergy were identified using root cause analysis. Comments and problems were marked with yellow and red tags, respectively. Furthermore, simulations using a stopwatch were carried out to assess throughput times of critical connections between operating units. After the analysis, the planning group redesigned the whole process according to the four defined key fundamentals.

The key values in the beginning of life process, especially family-centered care, were not up for compromise. These values were subsequently adopted by each of the six functional design groups planning the new Women's and Children's Hospital. The project coordinator and a Lean consultant worked with each of the groups to ensure the desired design methods and values were comprehended.

Functional planning was followed by layout planning alongside the architects. It was seen as favorable that the design team had the chance to alter the hospital layout. The whole staff was involved in commenting on the layout draft based on their experience with clinical processes.

### 3.4. Final Outcome of the Beginning of Life Process

One of the most beneficial structural changes in the developed functional model was the placement of the prenatal maternity ward, the labor and delivery room, and the NICU on the same floor and in immediate proximity to each other. This was done to enable synergies leading to decreased patient traffic and transfers. This would lead to better collaboration between the staff of different units and more continuity in care.

To ensure efficient use of space, functional triage models were suggested for both obstetric and neonatal admissions. When there is a triage space that allows staff to observe the mother on entering the hospital or the newborn after birth, it is more likely that they will be admitted to the most suitable ward.

The goal of the obstetric triage model was to combine pregnant women's emergency care process with delivery room admissions. It was planned for the triage to be located in immediate proximity to the prenatal maternity ward, but for either prenatal maternity ward or labor ward midwives to function as triage nurses. The elective maternity outpatient clinic was to be separate from the emergency care. All acute care was to proceed through a single pathway, in which a midwife was to admit the new patient. All telephone consultations from outside the hospital were to be taken in a centralized call center in the triage space, allowing mothers to be monitored from home before arriving at the hospital (e.g., in the latency phase). Mothers were to be assessed for care or follow-up needs and then either transferred from the triage to the labor and delivery room, to the prenatal maternity ward, or back home if labor was not in progress. Separating the emergency process was expected to decrease traffic in both the maternity outpatient clinic and the labor and delivery room. The labor and delivery room could therefore focus on mothers in active labor and delivery as the prenatal maternity ward would admit emergency patients. The rooms in the prenatal maternity ward were planned for one patient, but with an additional bed for the spouse according to the philosophy of family-centered care.

The inclusion of triage for newborns means that they can be monitored and treated in an adequately sized and equipped stabilization room located in the labor and delivery unit until the need for more extensive intensive care is identified or ruled out. Newborn infants may therefore avoid NICU admission if the symptoms resolve spontaneously and the initial laboratory tests or X-rays rule out the need for longer or more intensive monitoring or treatment. This saves staff costs as well as reserving space for specialized functions. The stabilization room was to have space for a mother's bed next to the newborn.

The integration of obstetric operating rooms for caesarean sections in the labor and delivery room makes the transfer to operation quicker, improves the safety of the mother and the newborn, and improves the efficient use of space as one stabilization room can be used for all newborns whether they are born vaginally or by caesarean section.

In the NICU, single family rooms were designed to accommodate the immediate family of an infant. Modifiable rooms in the NICU enable the care of both mother and infant in one room from admission until transition to home. Importantly, this decreases parent-infant separation for this vulnerable infant population and for parents who are developing an attachment relationship with their child. Parents and family members expressed their appreciation for a feeling of individuality (individual/customized welcome signs and guides), peacefulness (no other patients in the room), and protection of privacy (possibility of blocking visual contact to the room).

The innovations in hospital structures set new demands for the HC personnel, which need to be acknowledged for the benefits of the new structure to be realized. Parents and family members expressed their sensitivity to and the importance of staff attitudes and explained how the feeling of being welcomed affects their willingness to utilize hospital space and to participate in the care of their infant in the hospital. The staff needed to acquire new skills as the new process was different to the old practice, which comprised separate staff for mothers and infants and less parental involvement in the hospital care of their infant. In 2009 the H1 NICU developed and initiated a goal-oriented training program for the entire staff about delivering care in partnership with parents (Close Collaboration with Parents Training Program™) [16] to prepare staff for the new architecture.

The family rooms were to be located as close as possible to the central monitoring stations to minimize the distance between the staff and their patients. The central monitoring stations were seen as valuable for maintaining good teamwork between staff members. A curve-shaped layout was seen as functional for this purpose.

**Figure 2:**
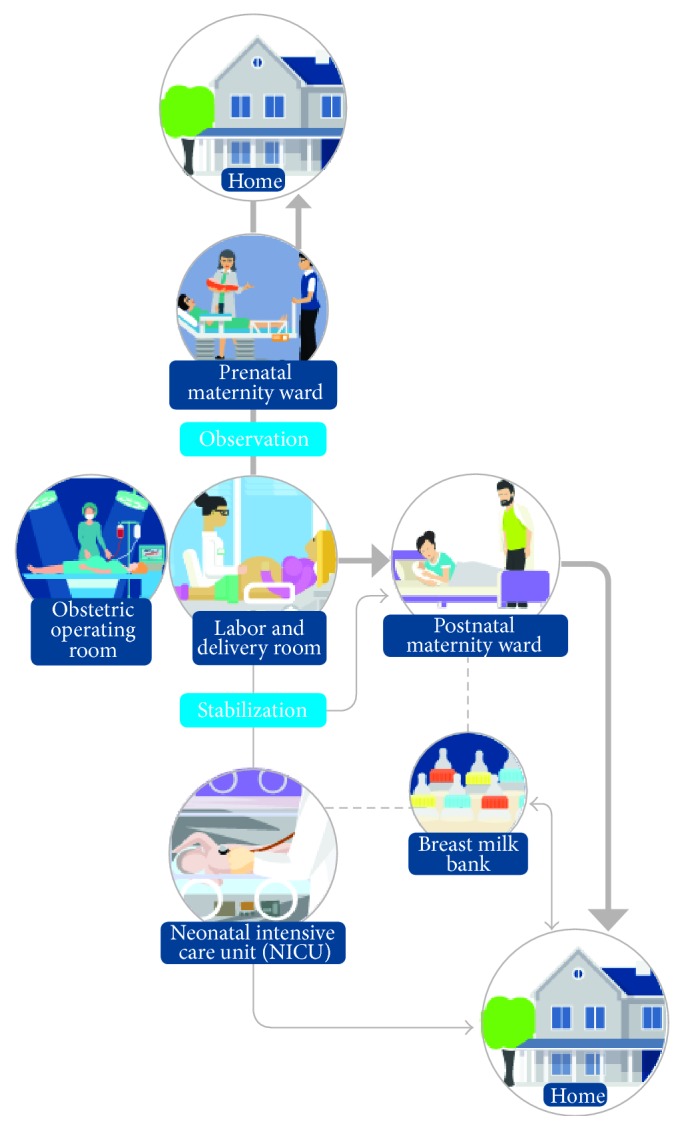
The newly developed H1 patient flow process model for the care of pregnant women and newborns. The new process minimizes parent-infant separation by, for example, locating more extensive stabilization for a sick newborn in the delivery room and by designing single family rooms with privacy and facilities for parents to stay overnight alongside their infant. Waste decreases and patient safety improves by, for example, decreasing the need for transitions between hospital wards and by preventing hospital infections with single family rooms. The delivery room and the NICU being in close proximity of each other and on the same floor increases synergy, especially in emergency situations.

The functional model for the beginning of life process can be seen in Figure 2.

The new model incorporated several desired benefits for patients, parents, and the hospital. The functional model for the beginning of life process simplified and streamlined patient flows and made them more flexible. The new model is believed to create a positive effect on infants' recovery and growth, as well as lowering patient and parent stress level due to increased physical and emotional closeness. Increased feelings of security and decreased levels of stress for the mother may also result from enabling the father's presence. If parents or support persons can be present within the hospital, the workload of hospital personnel might reduce in the long run:

- Parents are able to participate in the care of their infants.
- The spouse is able to participate in the care of the mother.
- The patient discharge process may become quicker.

### 3.5. Challenges Encountered

The original plans of The Beginning of Life Group were significantly altered during subsequent phases of the building project because the original budget estimate was reduced from €207 million to €158 million. This meant that major spatial reductions had to be made, which in turn decreased the functionality of the space. The required compromises impacted work processes due to heightened competition for space between the units in the hospital.

The design group wished they had been able to engage in earlier and closer contact and communication with the project architects. H1 cooperated with an architect company along the way but committed to them relatively late in the planning process, after funding for the architects had already been agreed upon. Optimally, the architects would have been included in the functional planning group. The functionality had to be explained and demonstrated separately to the architects, which significantly delayed the completion of sketches and also wasted time and effort in the planning process. Several FD group members were not satisfied with the initial sketches by the architects. Thus, the group decided to design the initial sketch of the new hospital independently, without the guidance of architects. After H1 committed to the architecture company, the exchange of information and the relationship gradually improved.

## 4. Discussion

### 4.1. What Has Been Learned?

Many functional weaknesses of the old hospital were identified by the leaders and the experienced staff, who were active in initiating preparation for the FD several years before the official mandate was given to prepare a functional plan for a new hospital. The process of learning about different solutions and their strengths, weaknesses, and relevance to the local project was time consuming. It was able to be done cost-effectively when combined with other professional travel during the early stages of the process.

When the official FD phase finally began, a large amount of documentation had been studied and several international visits and work periods in modern design settings had been carried out. The information had been discussed and reflected on in staff meetings. This facilitated the FD process and improved the design quality.

The planning group represented a wide range of professionals who had extensive experience in their roles and many international experiences to draw on for comparison. The inclusion of a patient representative in the planning group was seen as beneficial as her presence and active participation emphasized the patient perspective. Moreover, her contacts with key stakeholders were believed to facilitate the design process. It was seen as a significant weakness that the architects responsible for the layout were not chosen early on in the process, and thereby not included in the group. A real-time link with an architect company might have created solutions to problems which remained unseen in this process.

While clinicians' responsibilities can limit their participation in FD work groups [17], H1 allocated protected FD time for key persons, which proved to be a practical solution. Management needed to carefully weigh their options, such as which clinicians should be assigned to FD work and how intensely they should participate. Even though assigning an experienced HC professional to actively participate in an FD project may at first seem expensive, the investment is likely to benefit the design of the new building and lead to an increased capability of the clinicians to develop work processes suited to the new work environment.

When the total budget was decided, significant compromises had to be made. At this stage, the clearly determined key values and core goals were critical to keep in mind as priorities. In the planning phase, the priority level of each key value was carefully considered and established. Thus, H1 was able to develop structures for providing family-centered care, for supporting patient safety, and for implementing Lean principles and cooperation.

In Finland, HC is largely provided by the public sector. Thus, competition between hospitals differs from countries in which local, privately funded hospitals compete against each other. Lack of financial competition supports open knowledge sharing between hospitals both within and between countries. Humbleness and open-mindedness in sharing knowledge, FD information, and learning from and collaborating should be prioritized in areas where human lives are at stake [18]. In addition to better functional outcomes, information sharing is cost-efficient as it has the potential to prevent nonfunctional solutions and costly design mistakes. New forums and tools for learning and spreading vital FD information among HC practitioners are required [19].

The main focus for healthcare technology assessment (HTA) frameworks is to evaluate the properties and effects of a certain health technology, addressing the direct and intended effects and consequences of the technology. This helps in informed decision-making regarding health technologies. The HTA Core model is a methodological framework for production and sharing of HTA information [20]. It consists of a standardized set of HTA questions, recommends use of already existing guidance and guidelines, and includes a common reporting structure for presenting findings in a standardized format. Although it might have been useful to provide a proven international bridge between the state-of-the-art evidence-based research and the world of decision-making, it was not implemented due to the designers not being aware of the HTA Core model.

### 4.2. Family-Centered Care

As the top priority for the beginning of life process, family-centered care is likely to provide several benefits. First of all, single family rooms bring family members together and provide privacy, individuality, a sense of closeness, peacefulness, and protection, which have been shown to benefit all family members [21]. There is large, untapped potential for family members to improve the quality of care in both adult and neonatal care. Although the benefits may generate some economic savings for the hospital, the greatest advantages are likely to be seen in the improved wellbeing and satisfaction of the patients and their family members. In the case of newborn care, the benefits for the developing parent-infant relationship and for parents' psychological wellbeing are likely to improve the long-term outcomes of the infants.

Modifiable single family rooms in neonatal intensive care units support three of the key values of the planning: family-centered care, patient safety, and waste minimization. This model increases time efficiency as patients stay in one room for the duration of their hospital stay. The same care team also works with families for the entire time, which is favorable because lack of continuity is one of the most common complaints from parents [22]. This model also prevents hospital-acquired infections and thereby patient and staff safety [23]. Hospital-acquired infections prolong the hospital stay [24] and increase later developmental problems [25]. Therefore, the prevention of infection decreases both short- and long-term costs.

In order to successfully integrate parents in the care team it is advisable to prepare staff with training and practice in advance. In traditional intensive care units, parents have commonly had a passive role, primarily being recipients of information. To make the parents' role meaningful, they need to become information providers and active decision makers alongside staff [16]. Hospital design can emphasize the role of the family: parents need to have facilities that allow them to stay in the unit for several weeks, including overnight sleeping facilities, sanitary facilities, facilities for preparing meals and washing laundry, and rooms for socializing with other families in the unit. In addition, siblings need a play space separate to patient's room. The family rooms should be comfortable and let in daylight so that a long-term stay will be tolerable. Many hospitals have made the move to single family room units without an increase in staffing. Even if greater distances between patient beds and increased parental support requires more time input from staff, the investment is paid back when the parents become more independent in the care routines of their infant. In some hospitals, a move to the single family room model has led to shorter hospital stays [26] but this phenomenon has not been seen in all hospitals [27].

### 4.3. Patient Safety

A complex care process for mothers and infants includes many challenges. It is not necessarily known beforehand who requires more medical attention. Close collaboration and information sharing between staff that specializes in obstetrical and newborn care is crucial for success. The physical closeness of the maternal prenatal ward, the labor and delivery room, and the NICU is especially vital in emergency situations.

### 4.4. Lean Thinking

H1 utilized a unique method of conveying information to the functional work groups in the FD project: a project coordinator from the beginning of life process joined all of the other planning groups and divulged information and working methods. The working methods included value stream mapping, the Lean framework, and brainstorming sessions using extreme models as the starting point [12, 13]. Thus, utilizing this experienced specialist, who participated in each functional work group, proved crucial because the FD groups saved time in their working processes. This arrangement also enabled a standardized protocol to be carried out. The goal for each functional group was to set their own, customized FD priorities and incorporate their specialized needs into the building layout. The Lean ideology of streamlining patient care processes was taught to the user group representatives. Based on work flow processes, layouts for the new building were designed.

As the budget unexpectedly decreased during the project, significant spatial reductions had to be undertaken. Attempting to find answers, H1 FD personnel resorted to Lean hospital design, which suggested the centralization of HC work processes in order to utilize the space efficiently, with a focus on critical connections for patient flow [3]. H1 embraced these proposals and was successful in managing the reductions.

The triage model aimed to organize HC personnel among the care pathways in a way that would, ideally, suit the patient flow. By utilizing value stream maps, each step among the patient care pathway was optimized and the bottlenecks that were hindering the provision of efficient patient care were eliminated [28]. The key innovation was in combining care processes so that the route traveled by the patient was as quick and effortless as possible. A wide array of research supports centralizing and streamlining acute care processes to emphasize fluent patient flow [29, 30]. Multiprofessional assessment of the patient, with the possibility of observation time in an early phase, saves patient transfers as well as time later in the care process. Furthermore, home monitoring before delivery and early discharge lowers patient care expenses.

### 4.5. Future Challenges

Despite the vast majority of scientific evidence pointing to the benefits of family-centered care [31, 32], mixed opinions and uncertainty have prevailed in some HC institutions [33]. Thus, gaining management approval to adopt and implement solutions such as single family rooms may prove difficult. H1's Beginning of Life Group made calculations to convince the management of the benefits of initial investment for larger single family rooms. This was cumbersome as there is a lack of cost-benefit analyses related to single family room design.

## 5. Conclusions

In the wake of stifling pressure to create enhanced facilities for providing efficient HC, H1 FD personnel have demonstrated self-initiative and creativity in laying the groundwork for the FD process. Rigorous preparation has paid dividends and facilitated the FD process. Family-centered care, patient safety, Lean thinking, and cooperation were chosen by The Beginning of Life Group as the key values needed to achieve the core goals for the new hospital. This strategy was successful, as the four fundamentals have made a strong imprint on the FD process and have visibly guided it.

The FD based on functional planning is a good start. The ultimate quest lies in implementing the new functional schemes into the new facilities. Thus, the employees hold the keys to making the changes enabled by the new design. Implementing the functional principles among the staff is an ongoing process. Strong, determined, and insightful leadership should provide a solid foundation.

## Figures and Tables

**Table 1:** The key values defined by The Beginning of Life Group.

Priority	Value	Goal	Example
1	Family-centered care	Minimize parent-infant separation	Family rooms
2	Minimize waste	Create a Lean hospital	Modifiable rooms
3	Patient and staff safety	Improve hospital safety	Less patient transfers
4	Cooperation	Improve employee cooperation	Units closer together
